# Assessment of genetic diversity, tissue tropism, and antigenic properties of Grimsö betacoronavirus in Swedish bank voles (*Clethrionomys glareolus*)

**DOI:** 10.1016/j.onehlt.2024.100911

**Published:** 2024-10-09

**Authors:** Santiago Fernández Morente, Jinlin Li, Anishia Wasberg, Inês R. Faria, Elin Economou Lundeberg, Bo Settergren, Åke Lundkvist, Jiaxin Ling

**Affiliations:** a Zoonosis Science Center, Department of Medical Biochemistry and Microbiology, Uppsala University, Sweden; b Department of Medical Biochemistry and Microbiology, Uppsala University, Sweden; c Department of Infectious Diseases, Central Hospital of Kristianstad, Kristianstad, Sweden

**Keywords:** Betacoronavirus, Genetic diversity, Cross-reactivity, Tissue tropism

## Abstract

Zoonotic coronaviruses can transmit over species barriers and infect humans. To understand the zoonotic potential of a betacoronavirus, Grimsö virus (GRIV), we investigated the geographic distribution and tissue tropism of GRIV in Swedish bank voles (*Clethrionomys glareolus*), and the antigenicity of the nucleocapsid (N) protein. We screened the lung tissues from animals collected in the southern Sweden by RT-PCR with primers targeting the spike gene. Seven out of 74 animals were found to be positive. They are genetically close to GRIV from Grimsö, central Sweden. Positive rodents were studied for the tissue distribution of GRIV and GRIV RNA was mainly found in the respiratory tract. After three attempts of virus isolation were failed, we successfully established a Vero E6 cell line that stably expressed GRIV N protein, which has no cross-reactivity with patient serum containing antibodies against SARS-CoV-2, or with MERS-CoV. However, a low level of cross-reactivity to common cold coronaviruses was found, likely HCoV-OC43 or HCoV-HKU1, probably due to shared linear epitopes. With the high prevalence and the suggested respiratory transmission route, GRIV may have a high potential for spillover and cross-species transmission, and future serological screening of GRIV infections in domestic animals or humans will be needed.

## 1 Introduction

Rodents constitute over 43 % of all mammalian species and are important reservoirs for numerous zoonotic pathogens, including coronaviruses, hantaviruses, arenaviruses, cowpox, rotavirus, paramyxovirus and other emerging agents with pandemic potential [[1], [2], [3]]. Human activities, such as urbanization and globalization, have had severe impact on wildlife ecosystems and facilitated cross-species transmission of these pathogens [4]. Therefore, biomonitoring rodents and the pathogens they harbor is essential for effective pandemic preparedness.

One of the pathogens with high risk of emergence is coronavirus. Coronaviruses (CoVs) belong to the *Coronaviridae* family in the *Nidovirales* order and constitute a group of viruses with an unusually large RNA genome (ranging from 26 to 32 kb). Their genomes encode 16 non-structural proteins, including conserved enzymes such as the 3C-like protease (Mpro or 3CLpro), a papain-like protease (PLpro) and the RNA-dependent RNA polymerase (RdRp), which are responsible for the proteolysis and replication. The genome also encodes four principal structural proteins: the spike (S) protein, the envelope (E) protein, the membrane (M) protein, and the nucleocapsid (N) protein. The S protein binds to the host cell receptor and promotes the fusion of the virion with the cell membrane, whereas the E protein is an amphipathic small polypeptide that is divergent among the coronaviruses. The M protein is responsible for the virus spherical shape and the N protein binds the genomic RNA. CoVs are widely distributed in nature and carried by various mammalian and avian species. Rodents, together with bats, are the most plausible origin of the known pathogenic CoVs infecting human or domestic animals [5]. Despite being principally confined in their natural reservoir, due to the ever-evolving feature and continuous expansion of the host range, these CoVs can emerge and infect humans or domestic animals. Infection can cause gastrointestinal or respiratory symptoms, exemplified by cases seen for SARS-CoV, MERS-CoV, and SARS-CoV-2 in humans [6], as well as swine acute diarrhoea syndrome coronavirus (SADS-CoV) in pigs [7].

Based on the phylogenetic relationship, CoVs can be divided into four genera: mammals are mainly infected by alpha-CoV and beta-CoV, while gamma-CoV and delta-CoV infect birds [5]. The genus of beta-CoVs, consisting of four subgenera *Embecovirus*, *Merbecovirus*, *Nobecovirus,* and *Sarbecovirus*, comprise a range of CoVs of great importance. These include HCoV-OC43 and HKU1, both of which cause the common cold in humans, and SARS-CoV-2, causing COVID-19, MERS-CoV, and the previously emerging SARS-CoV. Additionally, several beta-CoV infect domestic animals, such as Bovine Coronavirus (BCoV), causing respiratory and enteric disease in ruminants [8], Canine Respiratory Coronavirus (CRCoV) in domestic dog populations, Porcine Hemagglutinating Encephalomyelitis virus (PHEV) causing acute gastroenteritis in farmed pigs, as well as Murine Hepatitis virus (MHV) in mice. MHV, the well-characterized prototype of rodent-borne beta-CoV, is a pathogen of *Mus musculus* that can cause a broad range of symptoms starting in the respiratory tract down to the enteric mucosa or demyelinating diseases in mice, depending on the organotropism of the MHV strains [9]. Interestingly, all aforementioned endemic coronaviruses belong to the group of lineages, or subgenus *Embecovirus*, which includes the most ancestral lineage of CoVs from rodents, suggesting that HCoV-OC43 and HCoV-HKU1 which are endemic in humans, and BCoV, CRCoV, PHEV, and MHV endemic in domestic animals, most likely originate from rodents as a primordial zoonotic reservoir [6]. Therefore, discovering novel beta-CoVs from wild rodent species to identify new potential zoonotic risks is an important and extensive task. Several virus-discovery studies have been performed, and many novel virus sequences have been published [10,11]. However, few studies have included functional assessments of the virus.

**Fig. 1:**
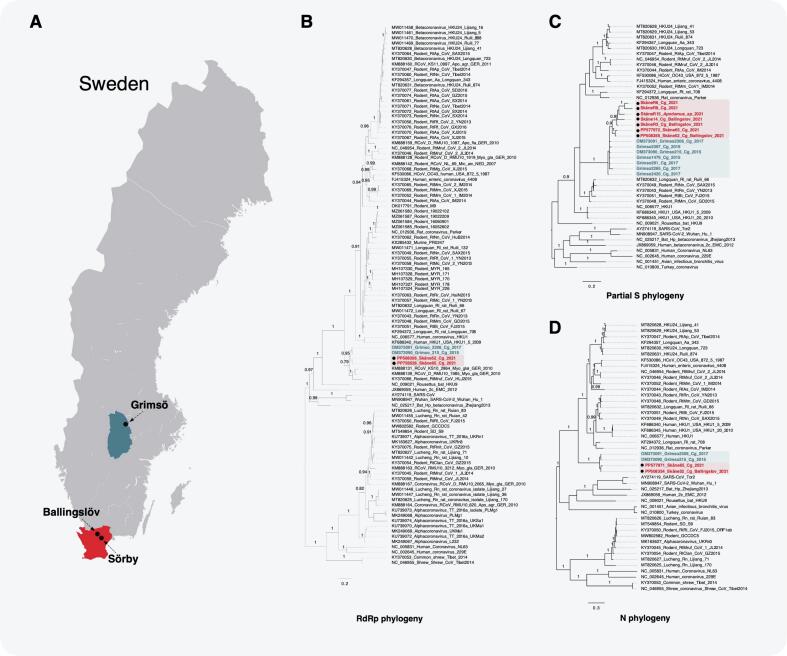
Phylogeographic distribution of GRIV in Sweden (A). Bayesian phylogenetic trees of GRIV based on the partial sequence of RdRp (B), partial sequences of S (C), and full length of N (D). GRIV strains from Grimsö were marked in blue and GRIV strains from Scania were marked in red. The obtained sequences from partial RdRp, full length of S, and N genes were deposited in Genbank. (For interpretation of the references to colour in this figure legend, the reader is referred to the web version of this article.)

Recently, we reported the discovery of a novel beta-CoV, Grimsö virus (GRIV), in Swedish bank voles (*Clethrionomys glareolus*) trapped in Grimsö in central Sweden (Fig. 1., Grimsö location— 59°43′N, 15°28′E) [12]. Based on full-genome sequencing, GRIV was classified as a beta-CoV within the subgenus *Embecovirus*. Expanding on our previous work, in this study, we aimed to understand the genetic diversity and tissue tropism of GRIV in Swedish bank voles, and the antigenic cross-reactivity of GRIV with CoVs pathogenic to humans.

## 2 Material and methods

### 2.1 RNA extraction and cDNA synthesis

Rodent samples were from the study by Ling et al. [13]. Briefly, rodent sampling was conducted using mouse snap-traps and Supercat vole traps in September 2020, May 2021, and September 2021 at three locations: Ballingslöv, Norra Sandby, and Sörby in Scania, Sweden. All samples were sent to laboratory through cold chain and lung tissues were harvested for the initial screening. Total RNA was extracted using the RNeasy mini kit (Qiagen, Hilden, Germany), followed by subsequent cDNA synthesis using the High-Capacity cDNA reverse transcription kit (Applied Biosystems, Vilnius, Lithuania) following the manufacturer's instructions.

### 2.2 Sequencing of RdRp, N and S genes

A primer walking strategy was used to sequence the RdRp, N and S genes. Primers were designed based on the GRP215–15 GRIV sequence (GenBank accession number OM373090) previously reported [12]. All PCRs were performed using Phusion Hot Start II High Fidelity (ThermoFisher, Vilnius, Lithuania) or AmpliTaq Gold (Applied Biosystems, Vilnius, Lithuania). Primer sequences are displayed in Table S1. Purified products were sequenced using Sanger sequencing (Macrogen, Amsterdam).

### 2.3 Tissue distribution of GRIV

To examine the tissue distribution of GRIV, solid organ tissues, and rectal and pharyngeal swabs were collected from rodents found to be positive by RT-PCR targeting the partial S-gene. Tissue materials were collected according to the physiological systems: respiratory, digestive, lymphatic and reproductive systems. To attempt virus isolation, approximately 100 mg of tissue was homogenized in 500 μL of DMEM (Gibco™, UK), and swab samples were stored in viral transfer media (VTM), containing HBSS supplemented with 2 % FBS, 100 μg/mL Gentamicin and 0.5 μg/mL Amphotericin B. The tissue suspension and VTM inoculums were vortexed, and 1:5 and 1:50 dilutions of the homogenates were used to inoculate Vero E6 cells, and Vero E6 cells expressing TMPRSS2 [14], followed by three blind passages as described earlier [15]. We collected supernatants from each passage from different cell models and cells were split until the third passage for final detection using RT-PCR targeting to the S gene, along with supernatant samples. All virus isolation attempts were performed in the Biosafety Level 3 (BSL-3) laboratory at ZSC.

### 2.4 Genetic characterization and phylogenetic analyses

To predict potential furin cleavage sites in the GRIV S protein sequence, the ProP program [16] was used, comparing the GRIV sequence to other coronaviruses with previously furin cleavage sites [17]. The genetic diversities of the RdRp, S, and N genes were calculated and compared to reference sequences. The phylogenetic analyses were performed as described earlier [12].

### 2.5 Generation of N protein overexpressing cell line

Due to restrictions on using genetically modified microorganisms at Uppsala University, we were only allowed to produce the GRIV N protein by then. Two separate methods for generating cells expressing the GRIV N protein were used: lentiviral transduction of Vero E6 and transient transfection of HeLa cells. The full length of the GRIV N gene from sample 52 was cloned into the plasmid pLVX-Puro vector (Clontech, CA, USA) hereby pLenti-flag-N, and the sequence was confirmed by Sanger sequencing.

In brief, lentivirus was produced in HEK293T by transfecting plasmids pLenti-flag-N, psPAX2 and pMD2.G using Lipofectamine 3000 (ThermoFisher Scientific, MA, USA) following the manufacturer's instructions. At 72 h post-transfection, supernatants containing lentiviral particles were collected. Viral particles were precipitated and concentrated using polyethylene glycol rotating overnight at 4 °C. Vero E6 cells were then infected with the harvested lentivirus for 1.5 h at 37 °C, 5 % CO2 and 6 mg/mL puromycin was added 24 h post-infection to select for transduced cells. Once stably growing, the cell line was examined for expression of N protein by using immunoblotting (IB) and an immunofluorescence assay (IFA) as described earlier [18].

The transient transfection of HeLa was performed using Lipofectamine 3000 where the cells were transfected with the pLenti-flag-N plasmid according to standard procedures. At 6 h post-transfection, the media was replaced with cell maintenance media. Cells were fixed with 100 % cold methanol 24 h post-transfection and analyzed for GRIV N expression using IFA.

### 2.6 Immunoblotting and immunofluorescence assay

For IB, lentiviral transduced Vero E6 cells and transfected HeLa cells were harvested, lysed and separated by SDS-PAGE gels, and subsequently transferred onto nitrocellulose membranes. The membranes were blotted with Anti-Flag M2 antibodies (Sigma-Aldrich, St. Louis, MO, USA), heart extracts of 7 positive samples, and a standard human serum panel [19], followed by HRP-conjugated secondary antibody (Bio-Rad Laboratories, Hercules, CA, USA). The hearts were harvested and stored in 500 μL of PBS. The samples were then vortexted and stored at +4oC overnight. The samples were then heat-inactivated at 60oC for 30 mins prior to performing IB and IFA.

For IFA, the cells were fixed with 100 % cold methanol on coverslips. After washing, the cells were permeabilized with 0.05 % Triton X-100, followed by blocking in 4 % BSA according to standard IFA procedures. Similarly, as for the IB, the fixed cells were incubated with the Anti-Flag antibody (1:500), rodent heart serum (1:5), or human serum (1:20) for 1 h at room temperature, followed by 1 h incubation with fluorescence-conjugated secondary antibody (1:1000). Slides were prepared in mounting buffer containing DAPI for nuclear visualization and later examined using a Nikon Eclipse 90i microscope.

## 3 Results

### 3.1 Genetic diversity of GRIV in Swedish bank voles

The rodents were collected in Scania between 2020 and 2021. In total, 74 rodent-like samples were collected and the morphological identification during the dissection revealed 48 bank voles (*C. glareolus*), 25 *Apodemus* spp.*,* and one *Sorex* spp. The lung tissues collected were screened for GRIV RNA by an RT-PCR targeting the partial S gene. We found seven GRIV-positive tissues, six from bank voles and one positive *Apodemus* spp. All positive samples were confirmed to be GRIV by sequencing, and the sequencing quality was enough for downstream analyses.

We later tried to obtain more sequences from these seven positive samples. However, partial RdRp sequences, the full length of the S gene, and the full length of the N gene could only be recovered from two samples – No. 52 and No. 65. Additionally, we employed a previously published pan-coronavirus RT-PCR targeting a conserved RdRp region but without any success [20], suggesting that GRIV is highly divergent from currently known CoVs.

Analyses of the sequenced genes revealed a new lineage of GRIV in Scania. Based on the partial RdRp gene, GRIV strains in Scania shared a 96.6 % nucleotide (nt) identity and a 98.7 % amino acid (aa) identity compared to the GRIV strains from Grimsö. GRIV in Sweden had the closest identity with the CoVs found in bank voles in Germany in 2010 (KS_2864 and RMU10_1985) and another CoV found in China, with 90.4–91.1 %/92.2–94.6 % and with 87.7 %/91.4 % identity at the nt/aa levels, respectively.

Based on the full length of the N gene, the diversity of GRIV strains in Scania showed 95 %/97 % nt/aa identity, respectively. Compared to the strains from Grimsö, the GRIV strains in Scania shared an identity of 86.7–95.5 % at the nt level and 90.5–96.8 % at the aa level. We also compared the sequence identity of GRIV (sample No. 52) with other pathogenic CoVs, with an identity (nt/aa) of 54.8 %/53.7 % to HCoV-OC43, 51.8 %/49.3 % to HCoV-HKU1, 30.3 %/21.9 % to HCoV-NL63, 28.9 %/20 % to HCoV-229E, 34.6 %/29.6 % to SARS-CoV, 35.5 %/28.3 % to SARS-CoV-2, and 34.6 %/29 % to MERS-CoV.

Regarding the S gene, the new lineage showed 98.9 %/98.8 % nt/aa identity within GRIV in Scania. Compared to the strains in Grimsö, GRIV strains in Scania shared an identity of 92.0–96.7 % at the nt level and 91.8–98.8 % at the aa level. Of the 21 amino acids mutated in the S protein, 2/3 were detected in the S1 domain, the domain responsible for the attachment to the cell receptor, a key determinant for cell entry. A furin cleavage site in the GRIV spike protein (RNKR) was predicted between domains S1 and S2 using the ProP program, obtaining a score prediction of 0.802 (a predicted site requires a score over 0.5, with the maximum being 1.0).

Finally, we analyzed the mutation pattern of the three sequenced genes separately and globally (Table S2). For GRIV N-gene, G > U and C > U are the most common transition and transversion. However, for the S gene and the partial RdRp gene, the displayed pattern shows that the most common transition is U > C. The most common transversion, U > A was only evident in the S gene, not the partial RdRp gene. Overall, U > C and G > U are the most common transition and transversion, respectively.

### 3.2 Phylogenetic analyses of GRIV

We further analyzed the phylogenetic relationship of GRIV strains from Scania with other beta-CoVs. Based on the Bayesian inference for the phylogeny of the partial RdRp gene, GRIV strains from both Scania and Grimsö clustered together. They fell into the basal lineage of the BetaCoV1 (lineage A), which includes two CoVs from bank voles in Germany and one CoV from grey red-backed voles (*Clethrionomys rufocanus*) in China [11]. To further understand the phylogenetic relationship of other Swedish GRIV genes, we analyzed the partial S gene and full-length N gene where a similar phylogenetic topology was found, where the Swedish GRIV strains from the two locations clustered together. The consistency of phylogenetic trees based on the partial RdRp, partial S, and full length of N genes suggested that no recombination events have occurred for GRIV.

### 3.3 Tissue specificity and tropisms of GRIV

**Fig. 2:**
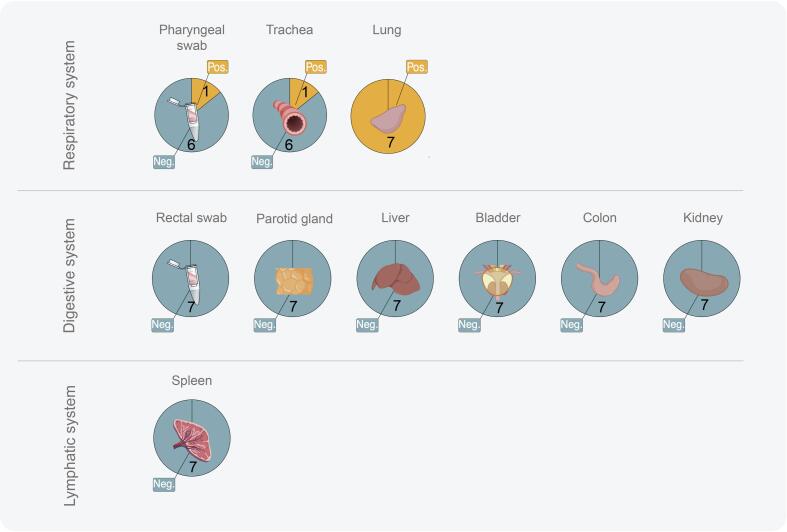
Tissue distribution of GRIV. All seven positive rodents for GRIV were further investigated for tissue tropisms. GRIV is mainly distributed in respiratory system.

To understand the shedding routes of GRIV, we dissected the GRIV positive rodents and proceeded with RT-PCR to investigate its tissue distribution. Meanwhile, to reduce free-thawing cycles, targeted tissue samples were added to Vero E6 cells for virus isolation. We found that one pharyngeal swab (R3) and one trachea (52) were tested RT-PCR positive for GRIV. This, together with the initial finding of positive lung tissues, suggests that GRIV is shedding through the respiratory system while not employing other systems, such as the digestive or lymphatic systems (Fig. 2 and Table S3).

### 3.4 Isolation attempts

For the rodents that were found to be positive in the initial screening, the solid tissue material and swabs were added to Vero E6 cells, and Vero E6 cells expressing TMPRSS2 for virus isolation. We did not detect any cytopathic effects or morphologic changes after three blind passages. We did not find any increased of viral genetic materials in the supernatant along with the increased number of passages nor were any viral genetic materials presented in the cells of the third passage, which implies that all the isolation attempts failed.

### 3.5 Expression of N protein in different cell lines

**Fig. 3:**
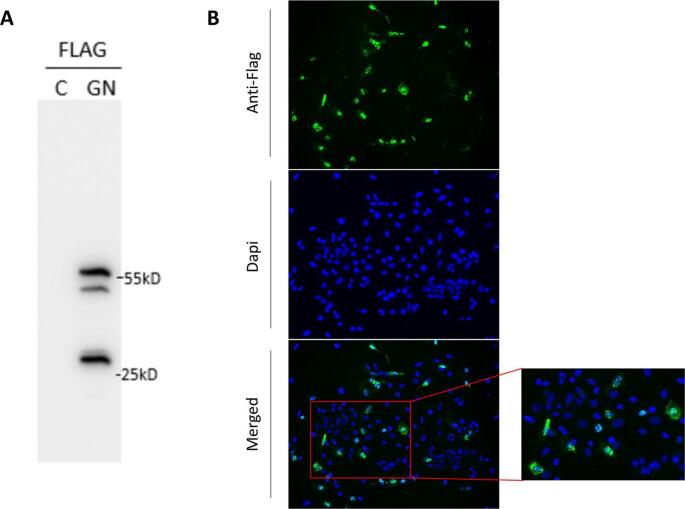
Production of the GRIV N protein in Vero E6 cells. After the transduction of Vero E6 cells using the lentivirus packaging system, the expression of N protein was examined by using both IB (A) and IFA (B). The molecular weight of GRIV N protein with Flag tag is about 55 kDa and two degraded/truncated N proteins were also detected in IB (around 30 and 50 kDa) from the cell lysates (A). The GRIV N protein is expressed in both cytoplasm and nucleus of Vero E6 cells.

**Fig. 4:**
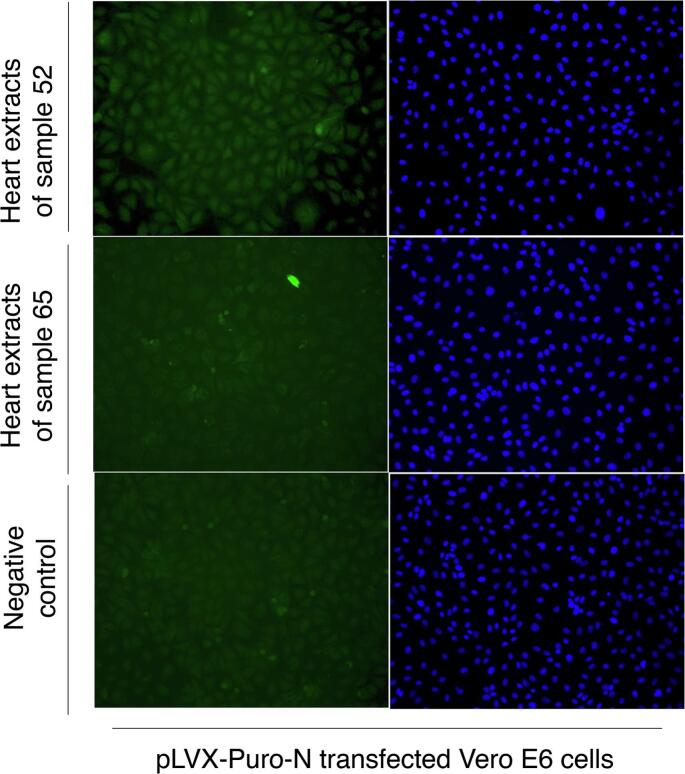
The GRIV N protein in Vero E6 cells was detected by IFA using positive rodent heart extracts.

We successfully cloned the N gene into the pLVX-Puro vector and examined the expression of the N protein in both HeLa and Vero E6 cells. In the HeLa cells, transient transfection was successful, and the nucleus was shown to be the primary location of the N protein. After transducing the Vero E6 cells using the lentivirus packaging system, we examined the expression of N protein using both IB and IFA (Fig. 3A and B). The predicted size of the N protein of GRIV is about 51 kilodaltons (kDa) [21] and the molecular weight of the protein with the Flag-tag was about 55 kDa as shown in Fig. 3A. High expression of the GRIV N protein was successfully achieved in Vero E6 cells, as indicated by staining with an anti-Flag Ab, with the nucleus as the main location of the protein (Fig. 3B). Additionally, we detected N protein expression in the Vero E6 cells using rodent heart extracts from GRIV-positive bank voles by IFA (Fig. 4) but not by IB. However, the expression level of the N protein decreased with increasing numbers of cell passages, suggesting that the GRIV N protein might be toxic for cells, as described by other similar studies [22].

### 3.6 Cross-reactivity of GRIV with other human pathogenetic CoVs

**Fig. 5:**
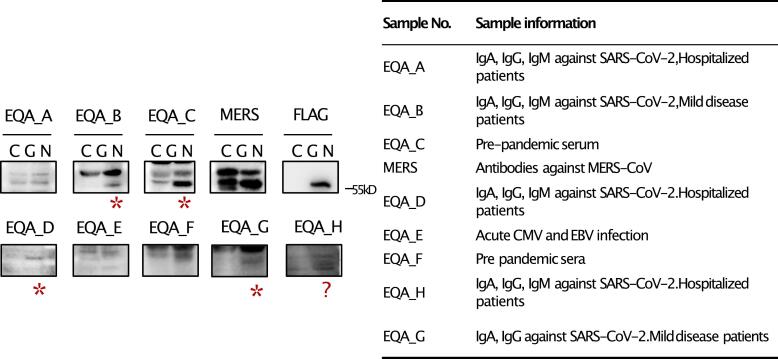
Detection of reactivity of GRIV N protein with serum samples containing HCoVs antibodies by immunoblotting. The asterisk indicates positive band as compared to the control group, and “?” indicates uncertainty of the result, where a weak band was detected.

We further analyzed IgG reactivity of a standard patient serum panel from the European Centre for Disease Prevention and Control (ECDC) consisting of a SARS-CoV-2 serological external quality assessment panel (EQA A-F) [19] and WHO MERS-CoV (NIBSC code 19/178) against the N protein of GRIV. We did not find any cross-reactivity by IFA, but we found some samples that showed reactivity to the GRIV N protein by IB (Fig. 5). Interestingly, not all the samples containing SARS-CoV-2 antibodies were positive for GRIV N. Sera containing MERS-CoV, CMV and EBV antibodies did not present cross-reactivities. Pre-pandemic serum (EQA_F) presented reactivity, strengthening the idea that there is no cross-reaction with SARS-CoV-2. At the same time, there is cross-reactivity to one or several of the human CoVs responsible for common colds (HCoV-NL63, HCoV-OC43, HCoV-HKU1, and HCoV-229E).

To understand why only some HCoVs show cross-reactivity with GRIV, we analyzed the results from the protein microarray conducted with the serum panel from Mogling et al., 2022 [19]. Table S4 contains the relevant information extracted from the microarray. Samples EQA_A, EQA_B, EQA_D, and EQA_H had a high level of antibodies against HKU1-S-T/ OC43-S-T (test results in an in-house protein microarray HKU1-S-T/ OC43-S-T are >4000 or > 3700 respectively, with a cut-off value for both methods being ≥10). As a result, GRIV seems to present cross-reactivity with at least one of them, most likely HCoV-HKU1 or HCoV-OC43, or possibly both.

As IB only detects linear epitopes, we aligned and compared the amino acid sequence of GRIV N protein (sample 52) with HCoVs N protein's reference sequences. The identity percentages of HCoV-OC43 and HCoV-HKU1 N proteins with the GRIV N protein are 56 % and 52 %, respectively, higher than HCoV-NL63 and HCoV-229E (Table S5). Furthermore, the OC43 and HKU1 N proteins share more linear epitopes with the GRIV N protein than NL63 and 229E (Fig. S2). This explains the cross-reactivity of GRIV with HCoV-OC43 and/or HCoV-HKU1 in IB.

## 4 Discussion

The emergence of coronaviruses in humans and domestic animals is increasing. Consequently, the monitoring and tracking of CoVs in nature to understand the host range and the potential of cross-species transmission is urgently needed. As primary reservoirs, rodents carry a broad range of CoVs, both in the subgenus *Luchacovirus*, genus *Alphacoronavirus*, and in the subgenus *Embecovirus*, genus *Betacoroanvirus* [23]. Recently, we discovered a novel beta-CoV, GRIV, in Swedish bank voles with an overall prevalence of 3.4 %. Furthermore, this virus seems to have a broad geographic distribution, and is not only found in central and southern Sweden but also elsewhere in Eurasia, as two CoVs from bank voles have been found in Germany, and one CoV from *C. rufocanus* has been reported from China (Fig. 1A). This study provides up-to-date knowledge of the genetic diversity, tissue tropism, and antigenic properties of the N protein of GRIV.

In this study, we further found a new lineage of GRIV in bank voles that have been sampled in Scania, 2020–2021, which provided additional evidence that bank voles are the primary hosts for GRIV, and that there is a long evolutionary relationship between bank voles and GRIV. We found that six out of 48 bank voles (12.5 %) and one out of 25 *Apodemus spp* were positive for GRIV. In total, our results suggested that the prevalence of GRIV is 9.46 % (7 positive cases out of 74 animals), with a 95 % confidence interval of 3.89 %–18.52 %. The positive *Apodemus spp* implies a spillover event of GRIV into another rodent species. We aimed to recover more sequences out of these seven GRIV-positive rodent samples, and the full lengths of S and N genes, and partial RdRp genes were successfully recovered from two samples 52 and 65. The results suggested that these two rodents probably had active infections of GRIV, in contrast to other samples e.g. SkåneR3_Cg_Ballingslov_2021, which was positive only by the partial S RT-PCR. However, a larger sample size with more detailed ecological data will allow us to understand more about infection ecology of GRIV in *C. glareolus.*

The genetic diversity of GRIV allowed us to examine the substitution pattern. We found C > U to be the main transition and G > U the main transversion, a common feature among CoVs in contrast to other positive-sense RNA viruses [24], which was only observed in the N gene of GRIV. The S gene, RdRp gene and all the sequences displayed different patterns (Table S2). As Forni et al. showed, the frequencies of the common mutation patterns mentioned tend to decrease as the divergence of the coronavirus increases. Therefore, GRIV included in this study might be highly divergent as the overall mutation frequencies of the studied genes do not resemble the general pattern, with U > C as the most common mutation. However, the N gene appears less divergent, resembling the common substitution pattern.

With the aim of isolating the GRIV virus, different cell lines were used for isolation attempts using all types of tissue materials containing GRIV viruses. All isolation attempts failed, even including Vero E6 cells expressing cleavage enzyme TMPRSS2, which has been approved as a functional receptor for HKU1, one of the closest relatives of GRIV [25]. Furthermore, our results suggested that GRIV mainly infects the respiratory system, as GRIV RNA was found in seven lungs, one trachea and one pharyngeal swab sample, indicating that excretion of GRIV is likely made through the respiratory tract. Considering the high population density of bank voles and the respiratory transmission route of GRIV, this virus seems to have a high potential for spillover and to infect other animals. In the future, respiratory cells or other types of cells from bank voles can be used for isolation attempts. Additionally, employing pseudotyped systems bearing the S or HE genes could be useful for pre-screening host cells to assess their permissibility prior to isolation, as described in [26].

To understand the capacity of cross-species transmission of GRIV, we planned to study the major virulence factors, and the S and hemagglutinin-esterase (HE) genes were planned to be studied. However, due to the regulation and debate on gain-of-function experiments for the pathogens with pandemic potential, an in vitro study was unfortunately not possible. Instead, prediction analyses have been used to understand the functions of the S and HE genes. We found that GRIV contains a furin cleavage site in the S protein, similar to most rodent-borne CoVs [27]. Furin proteases activate the spike protein from CoVs and facilitate the entrance of the virus into the cell. The presence of a furin cleavage site in the spike amino-acid sequence might be implicated in pathogenesis and transmission [17,27]. Additionally, the HE gene is usually present in rodent-borne CoVs of the *Embecovirus* subgenus, which has the dual functions of a binding domain and a receptor-destroying domain to help the virus attach to or release from the host cells. To further predict a potential carbohydrate-binding protein, the GRIV HE protein amino acid sequence (GenPept accession No. USS99156) was searched using UniLectin (https://unilectin.unige.ch/predict/) [28] and resulted in a score of 0.503, suggesting that the entry of GRIV into cells is probably mediated by glycan-binding. However, all these results are solely based on predictions using the newly sequenced genome of GRIV. Therefore, we hypothesize that GRIV uses sialic acids or other cell adhesion molecules to gain entry into the cells, similarly to other CoVs in this group, with MHV using CEA cell adhesion molecule 1a (CEACAM1a), and HCoV-HKU1, HCoV-OC43, and BCoV-ENT using sialic acids as (co-)receptors [29].

To develop a serological test for GRIV, we established a Vero E6 cell line that can stably express the N protein of GRIV with a Flag-tag, as the N protein of CoVs has a strong antigenicity and induces host immune responses during the infection. We found that the N protein of GRIV localizes in both the cytoplasm and nucleus, which has been observed similarly for other CoVs [22], and the functions of the N protein can explain this [30]. However, the expression of the protein decreased with the number of cell divisions, probably due to impairments during the cell division, as previously reported for other nucleocapsid proteins from other coronaviruses [22,31].

We later examined the cross-reactivity of GRIV and other pathogenic human CoVs using standard human serum panels. We did not find any cross-reactivity to GRIV N by using IFA. By IB, we found that there was a weak cross-reactivity of GRIV with at least one of the human CoVs responsible for common colds, i.e. HCoV-NL63, HCoV-OC43, HCoV-HKU1 or HCoV-229E. Based on the antibody levels of the different HCoVs causing common cold, we found that the GRIV N protein can react with the serum having higher levels of antibodies against HCoV-OC43 or HCoV-HKU1, as compared to SARS-CoV-2, MERS-CoV, HCoV-NL63, and HCoV-229E. This again agreed with the fact that GRIV N protein shares around 52–56 % of amino acid sequence identity with HCoV-HKU1 and HCoV-OC43, having the highest amino acid identities compared to the other HCoVs. The N protein has three different and conserved domains: The N terminal domain (NTD), the C terminal domain (CTD), and an intrinsically disordered region, which separates the two previous domains, known as the linker region (LKR) [30]. When comparing HCoV-OC43 and HCoV-HKU1 N protein domains [32] with the GRIV N protein, conserved linear epitopes are located within CTD and NTD rather than in LKR, which shows much lower identity (Fig. S1). In the future, we will test whether GRIV has cross-reactive epitopes with other CoVs of veterinary medical importance.

## 5 Conclusions

This study contributes to a better understanding of the genetic diversity, tissue tropism, and antigenic property of GRIV carried by Swedish bank voles. The presence of a furin cleavage site and a variable region detected in the S1 domain of the spike protein suggest a high potential of spill over or cross-species transmission. Additionally, there are no clear serological cross-reactions between GRIV N and pathogenic HCoVs, and therefore, the developed method can be further optimised for high throughput screening of GRIV in domestic animals and humans.

## Funding

J. Ling is funded by the Swedish Research Council (VR: 2022–03219), Åke Wibergs stiftelse (M22–0168 and M23–0189) and Carl Tryggers Stiftelse (CTS 23:2983). We thank the funding resources from the European Union's Horizon 2020 research innovation program under grant no. 874735 (VEO), from The Swedish Research Council (VR: 2017–05807) and by SciLifeLab, Pandemic Laboratory Preparedness (*Re*-LPP1–005).

## Ethics statement

Rodent sampling was performed according to required permits, including approval by the Animal Experiment Committee, Lund (reference 5.8.18–02281/2020), permission to handle laboratory animals from the Swedish Board of Agriculture (reference 5.2.18–14,256/2019), and a hunting permit by the Swedish Environmental Protection Agency (reference NV-02812-20). The rodents were placed on dry ice and sent via express postal service to the Zoonosis Science Centre (ZSC) in Uppsala, where they were stored at -80 °C until analyzed.

All sequences generated in this study have been deposited in the Genbank with accession numbers: PP577972, PP508355 (full length of spike gene for Skåne52 and 65), PP508356, PP756526 (partial RdRp gene for Skåne52 and 65); PP577971, PP508354 (full length of N gene for Skåne52 and 65).

## CRediT authorship contribution statement

**Santiago Fernández Morente:** Writing – review & editing, Writing – original draft, Investigation, Data curation. **Jinlin Li:** Writing – review & editing, Writing – original draft, Supervision, Methodology, Funding acquisition, Conceptualization. **Anishia Wasberg:** Writing – review & editing, Visualization, Investigation, Formal analysis. **Inês R. Faria:** Writing – review & editing, Visualization, Investigation, Formal analysis. **Elin Economou Lundeberg:** Writing – review & editing, Resources, Investigation. **Bo Settergren:** Writing – review & editing, Resources, Investigation. **Åke Lundkvist:** Writing – review & editing, Supervision, Project administration, Funding acquisition. **Jiaxin Ling:** Writing – review & editing, Writing – original draft, Supervision, Methodology, Funding acquisition, Conceptualization.

## Declaration of competing interest

The authors declare that they have no known competing financial interests or personal relationships that could have appeared to influence the work reported in this paper.

## Data Availability

Data will be made available on request.
