# Supplementary material for: Assessment of genetic diversity, tissue tropism, and antigenic properties of Grimsö betacoronavirus in Swedish bank voles (Clethrionomys glareolus)
Source: One Health. 2024 Oct 9;19:100911. doi: 10.1016/j.onehlt.2024.100911 (PMC11980623; doi:10.1016/j.onehlt.2024.100911)

**Supplementary**

**Table S1**. Primers used in primer walking experiments

1. **N protein:**

| Region (bp) | Reaction | Forward primer (5’- 3’) | Reverse primer (5’- 3’) | Producto length (bp) |
| --- | --- | --- | --- | --- |
| 29625 to  30439 | 1 | ATGTCTCTTATCCCAGGTC | TTGTACTCCAGGTGAGC | 815 |
| 30395 to  31020 | 2 | ATCCTCTAGCAGAGCAGCTT | TCAAAGTCTTTCTATTTGTGG | 626 |

1. **S protein:**

| Region (bp) | Reaction | Forward primer (5’- 3’) | Reverse primer (5’- 3’) | Product length (bp) |
| --- | --- | --- | --- | --- |
| 23832-24622 | 1 | TGCCCTACTGTTGACACGAC | TCACGCTCCAAAACACAATCG | 790 |
| 24587-25226 | 2 | TGCTCCGGAAAAAGGCGAT | AGGTAGCGCAAAACGATCAAC | 639 |
| 25002-25663 | 3 | CGATCAAAGTCCAATCTACCTGA | TTAGCCTGGGCATACAAGCG | 661 |
| 25597-26205 | 4 | AGCCTGGTGCTTGTACTGAT | CACAGAAACCATCACCAAGCG | 608 |
| 26160-26859 | 5 | TCCTTTGTCAAGCGTTGTGC | ATGCGGCACCAGCAGTATAA | 709 |
| 26767-27455 | 6 | ACCTCTTATGCACGCAGACC | ACCAGTACCATCAGGCAAACA | 688 |
| 27425-28179 | 7 | CCCAGGGTTATGTTTGCCTGA | TTTGAGAAAGAGCACGCCTCA | 754 |

1. **RdRp region:**

| Region (bp) | Reaction | Forward primer (5’- 3’) | Reverse primer (5’- 3’) | Product length (bp) |
| --- | --- | --- | --- | --- |
| 14746-15716 | 1 | GTTTATGATGGTGGTTGTATTC | ACGTCATTTTCGACCCAACAC | 970 |
| 14855-15716 | 2 | CTTTGTCTTTTGAAGAACAAG | ACGTCATTTTCGACCCAACAC | 861 |

**Table S2**. Transition and transversion mutations among GRIV genes

|  |  | Gene | S | N | RdRp | Overall |
| --- | --- | --- | --- | --- | --- | --- |
| Mutation | Transition | C > U | 25 % | 21 % | 21,5 % | 23,3 % |
|  |  | U > C | 30,8 % | 17,7 % | 35,8 % | 27,6 % |
|  |  | A > G | 9,2 % | 19,4 % | 7,1 % | 11,9 % |
|  |  | G > A | 11 % | 11,3 % | 14,3 % | 11,4 % |
|  | Transversion | G > U | 5,8 % | 11,3 % | 7,1 % | 7,6 % |
|  |  | U > G | 3,3 % | 1,6 % | 0 % | 2,4 % |
|  |  | U > A | 6,7 % | 8,1 % | 7,1 % | 7,1 % |
|  |  | A > U | 3,3 % | 6,4 % | 7,1 % | 4,8 % |
|  |  | C > G | 0’8 % | 0 % | 0 % | 0,5 % |
|  |  | G > C | 0 % | 0 % | 0 % | 0 % |
|  |  | C > A | 3,3 % | 1,6 % | 0 % | 2,4 % |
|  |  | A > C | 0,8 % | 1,6 % | 0 % | 1 % |
|  |  | Total NO. Of mutations | 120 | 62 | 28 | 210 |

**Table S3**. Summary of serological and RT-PCR results from 7 rodent samples positive for GRIV

| Sample No. | Species | Year | Lab analyses | | | | | | | | | | | | |
| --- | --- | --- | --- | --- | --- | --- | --- | --- | --- | --- | --- | --- | --- | --- | --- |
|  |  |  | Serology | | RT-PCR | | | | | | | | | | |
|  |  |  | IFA | IB | Lung | Pharyngeal swab | Trachea | Lung | Rectal swab | Parotid gland | Liver | Bladder | Colon | Kidney | Spleen |
| R6 | *C.glareolus* | 2021 | Neg | Nd | Pos | Neg | Neg | Pos | Neg | Neg | Neg | Neg | Neg | Neg | Neg |
| R8 | *C.glareolus* | 2021 | Neg | Nd | Pos | Neg | Neg | Pos | Neg | Neg | Neg | Neg | Neg | Neg | Neg |
| R15 | *Apodemus.spp* | 2021 | Neg | Nd | Pos | Neg | Neg | Pos | Neg | Neg | Neg | Neg | Neg | Neg | Neg |
| 14 | *C.glareolus* | 2021 | Neg | Nd | Pos | Neg | Neg | Pos | Neg | Neg | Neg | Neg | Neg | Neg | Neg |
| R3 | *C.glareolus* | 2021 | Neg | Nd | Pos | Pos | Neg | Pos | Neg | Neg | Neg | Neg | Neg | Neg | Neg |
| 65 | *C.glareolus* | 2021 | Pos | Nd | Pos | Neg | Neg | Pos | Neg | Neg | Neg | Neg | Neg | Neg | Neg |
| 52 | *C.glareolus* | 2021 | Pos | Nd | Pos | Neg | Pos | Pos | Neg | Neg | Neg | Neg | Neg | Neg | Neg |

Neg: negative result; Pos: positive result; Nd: not done.

**Table S4.** IgG titers detected by in-house-protein micro-array in serum panel used for GRIV cross-reactivity experiment. GRIV N protein IB is associated with each serum titers. Cut-off value ≥ 10. (Extracted from Mogling et al., 2022).

|  | HCoVs IgG titers detected | | | | | GRIV N protein IB result |
| --- | --- | --- | --- | --- | --- | --- |
| SERUM | NL63-S-T | 229E-S-T | OC43-S-T | HKU1-S-T | SARS-CoV-2-S-T |  |
| EQA-A | 2302 | 1603 | 7101 | 9775 | >14580 | _ |
| EQA-B | 1753 | 1382 | 3740 | 5271 | 6908 | + |
| EQA-C | 1264 | 1227 | 1272 | 570 | 10 | + |
| EQA-D | 1640 | 862 | 6496 | 4202 | 8800 | + |
| EQA-E | 1247 | 768 | 730 | 547 | 10 | _ |
| EQA-F | 1075 | 1005 | 1019 | 487 | 10 | _ |
| EQA-G | 1823 | 1460 | 1786 | 1716 | 2754 | + |
| EQA-H | 1597 | 816 | 3783 | 4198 | 5016 | + |

**Table S5.** Percentage of identity between GRIV N protein (sample 52) and HcoVs N proteins from reference genomes. Proteins ID are shown in brackets

| HCoV | NL63 (YP_003771.1) | 229E (NP_073556.1) | OC43 (YP_009555245.1) | HKU1 (YP_173242.1) | SARS-CoV-2  (YP_009724397.2) |
| --- | --- | --- | --- | --- | --- |
| % identity between N proteins | 42 | 31 | 56 | 52 | 35 |

**Figure S1**. Alignment of the two HCoV N proteins with higher grade of identity with GRIV N protein (HCoV-OC43 and HCoV-HKU1). Red residues show conserved linear epitopes. Blue residues show fragments with no gaps. Grey residues and grey lines (-) indicate the existence of a gap. Between brackets are compartmentalize the three different domains of N protein, showing that the vast majority of identity is located within NTD and CTD domains.

1. GRIV (upper row) and HCoV-OC43 (lower row) alignment. Green line demarcates **NTD** domain. Purple lines demarcates **CTD** domain. LKR domain is located between green and purple lines.


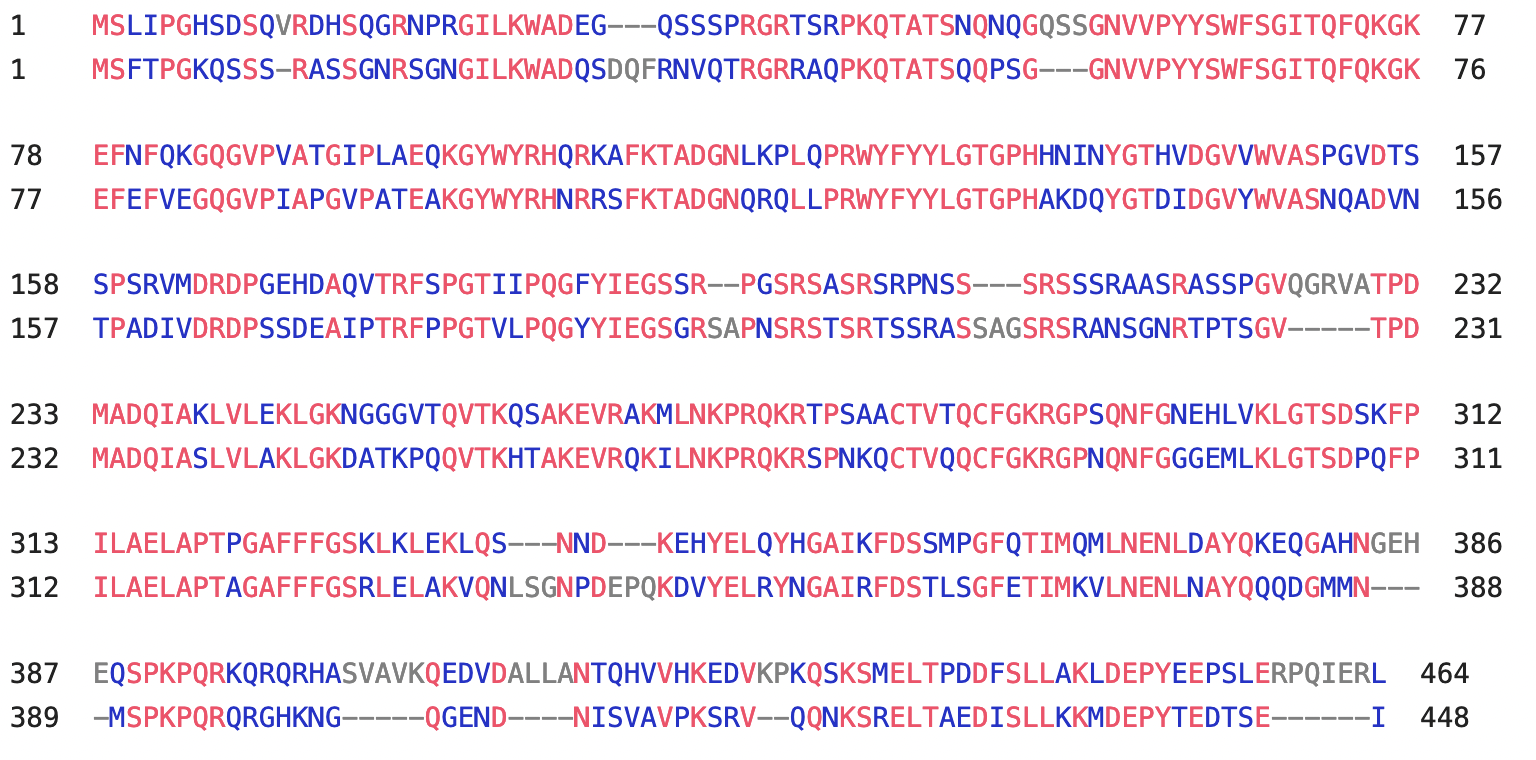


1. GRIV (upper row) and HCoV-HKU1 (lower row) alignment. Green line demarcates **NTD** domain. Purple lines demarcates **CTD** domain. LKR domain is located between green and purple lines.


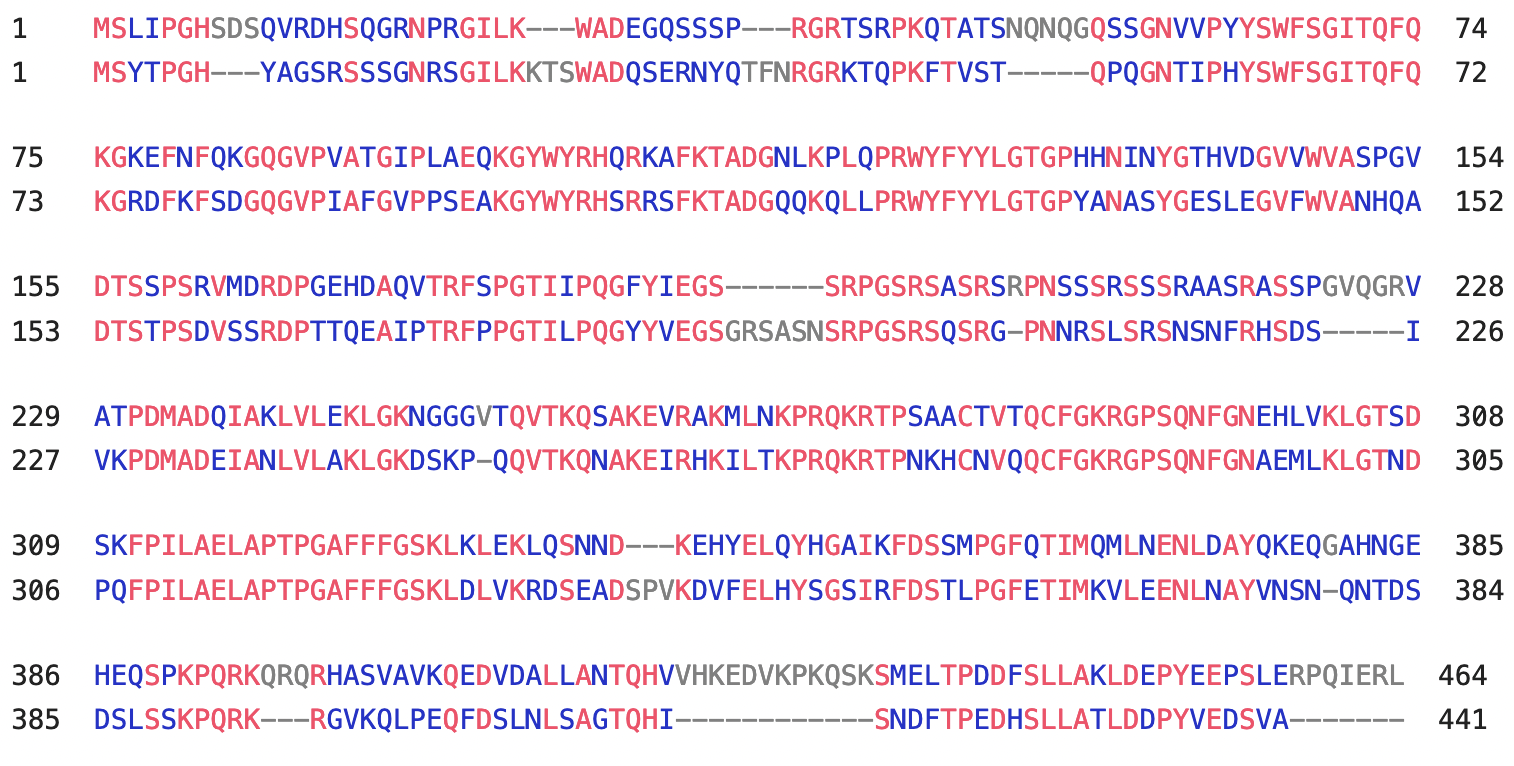


**Figure S2**. Alignment of GRIV N protein with different HCoVs N proteins. Red residues show conserved linear epitopes. Blue residues show fragments with no gaps. Grey residues and grey lines (-) indicate the existence of a gap.

1. GRIV (upper row) and HCoV-NL63 (lower row) alignment:


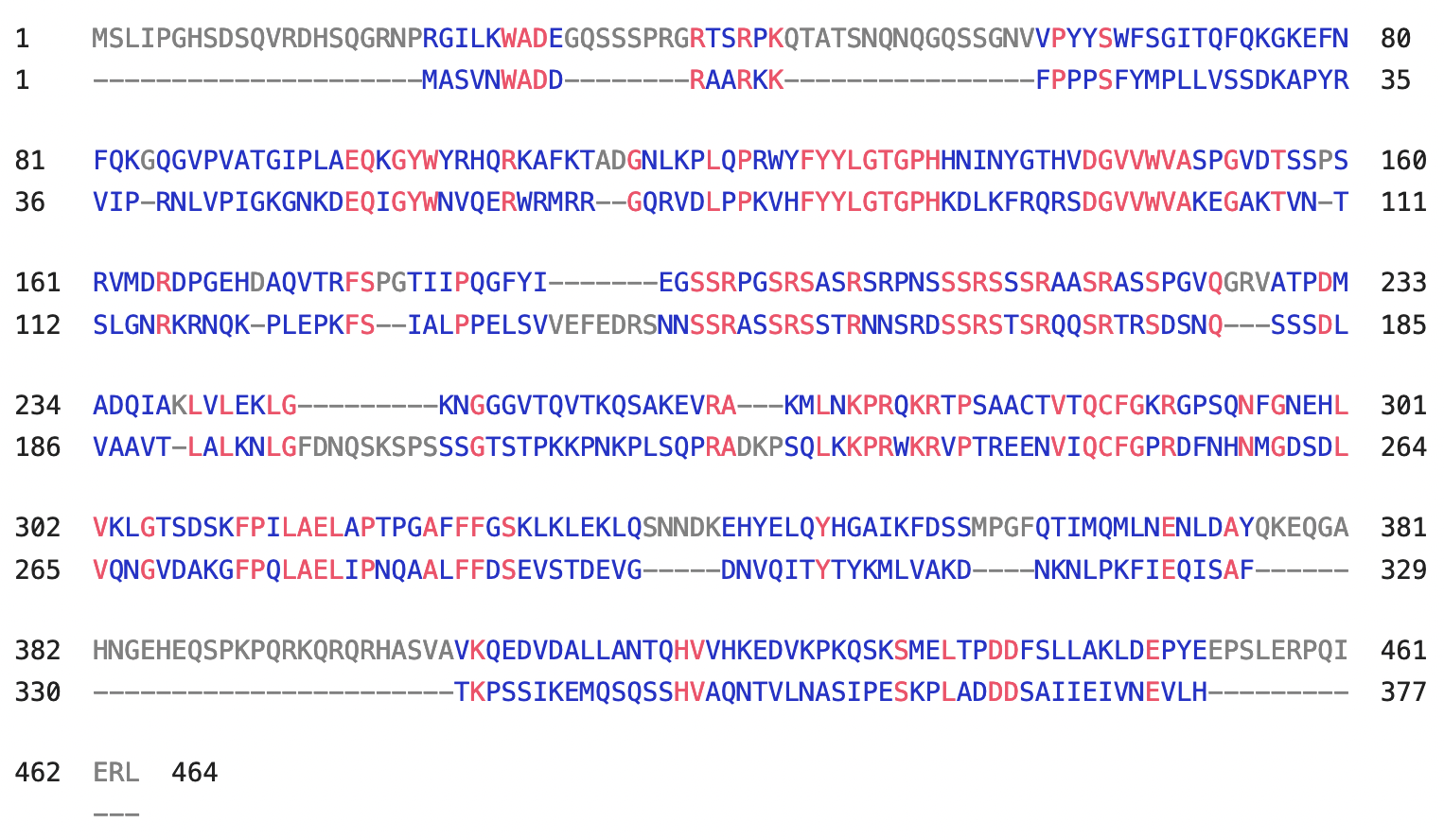


1. GRIV (upper row) and HCoV-229E (lower row) alignment:


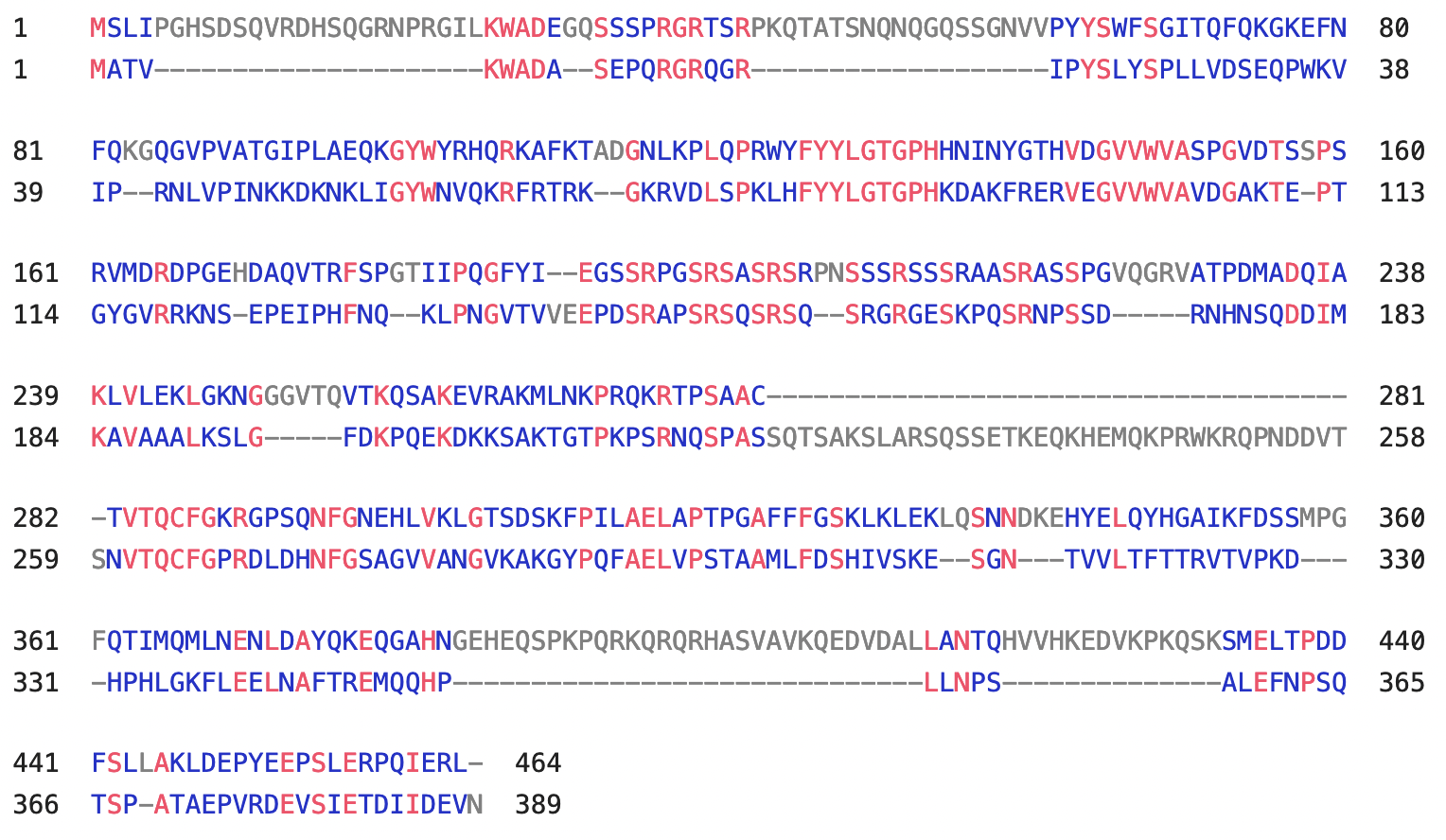


1. GRIV (upper row) and HCoV-HKU1 (lower row) alignment:


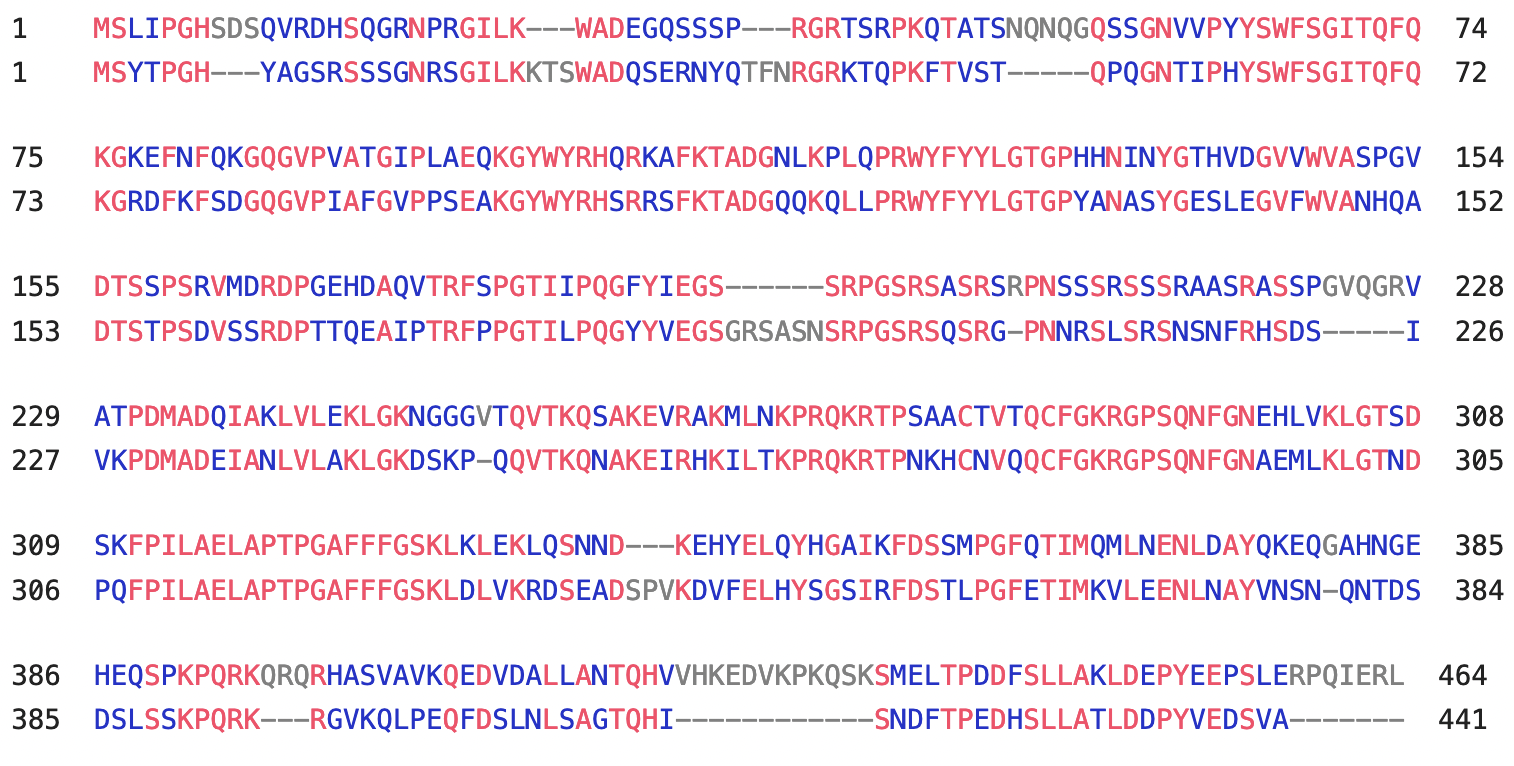


1. GRIV (upper row) and HCoV-OC43 (lower row) alignment:


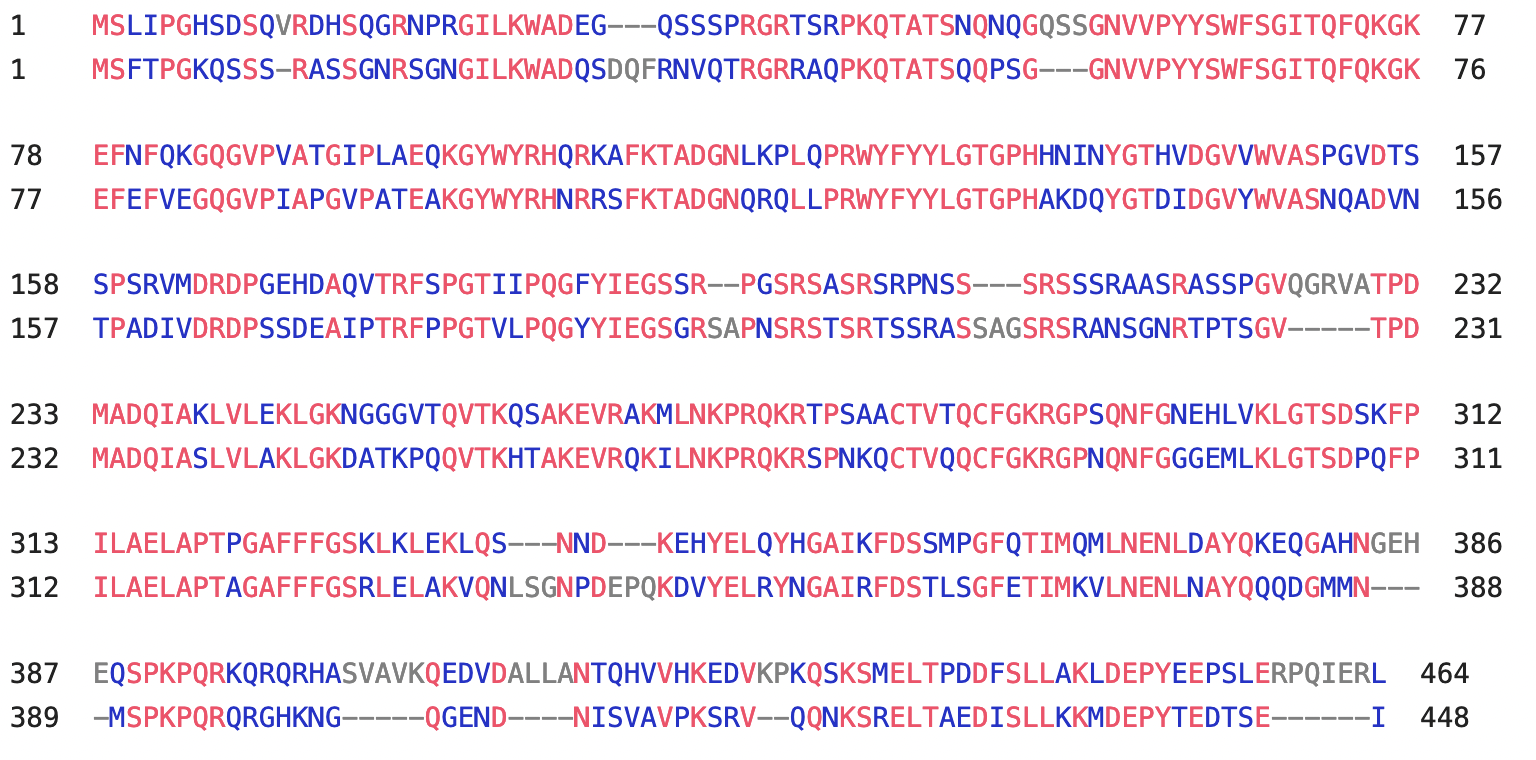


1. GRIV (upper row) and SARS-CoV-2 (lower row) alignment:


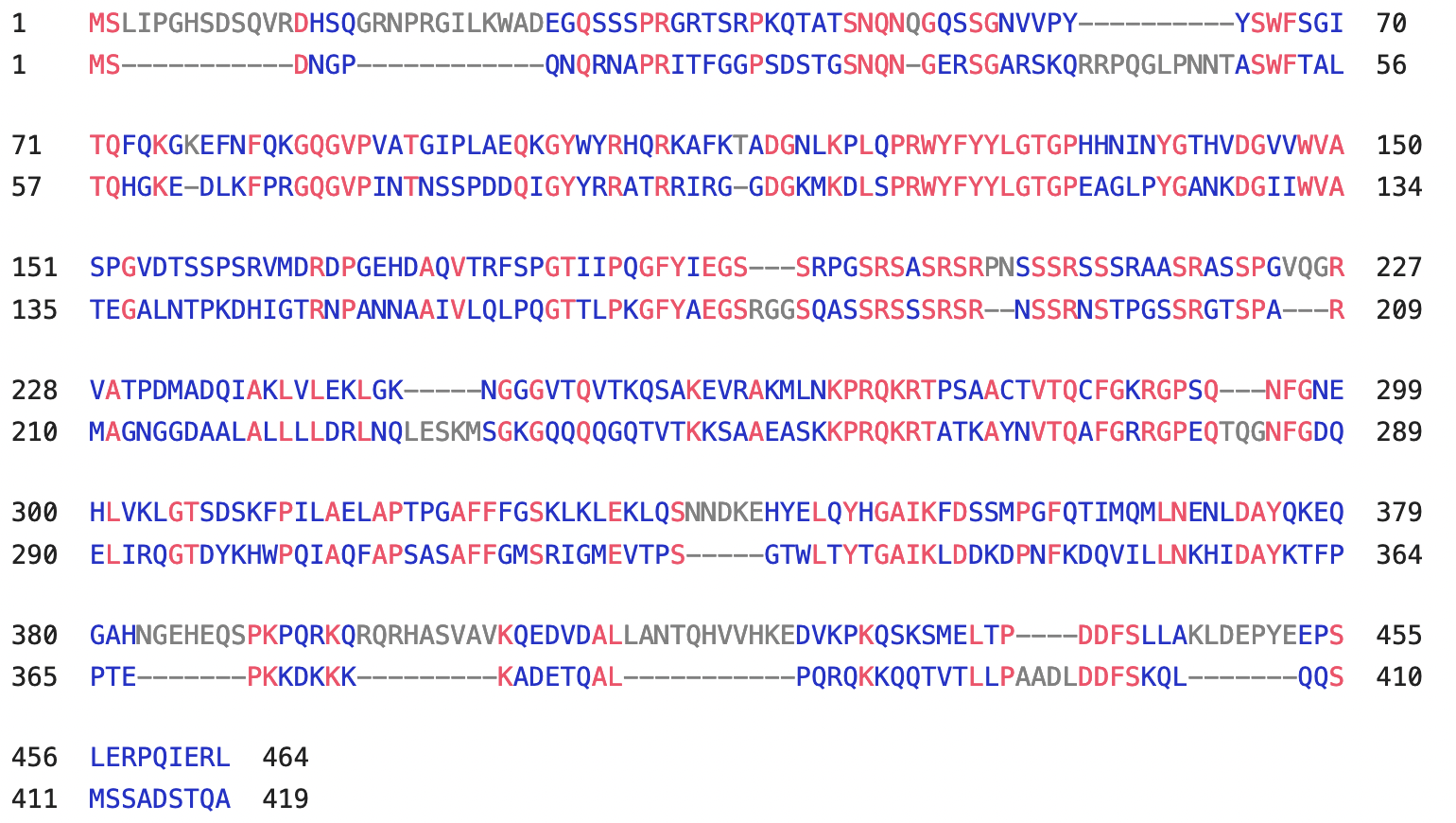

Supplement: Supplementary material [file mmc1.docx]
